# Supplementation of *Lactobacillus plantarum* (TCI227) Prevented Potassium-Oxonate-Induced Hyperuricemia in Rats

**DOI:** 10.3390/nu14224832

**Published:** 2022-11-15

**Authors:** Chih-Yu Chien, Yu-Jou Chien, Yung-Hao Lin, Yung-Hsiang Lin, Shu-Ting Chan, Wei-Chun Hu, Han-Fang Wu, Chi-Fu Chiang, Chin-Lin Hsu

**Affiliations:** 1 Department of Nutrition, Chung Shan Medical University, Taichung 40201, Taiwan; 2 Baiyuete Limited Company, Shanghai 200071, China; 3 Research & Design Center, TCI Co., Ltd., Taipei 11472, Taiwan; 4 Department of Nutrition, Chung Shan Medical University Hospital, Taichung 40201, Taiwan

**Keywords:** hyperuricemia, *Lactobacillus plantarum*, microbiota, uric acid

## Abstract

Hyperuricemia (HC) is one of the important risk factors for gout, arteriosclerosis, and cardiovascular disease. Animal studies have shown that *Lactobacillus plantarum* can improve microbiota and immune regulation, as well as inhibit uric acid production. However, it is not clear whether *L. plantarum* can improve HC and intestinal microbiota. We used potassium oxonate (PO) to induce HC in male SD rats and then treated them with *L. plantarum* TCI227 in a dose-dependent manner (HC + LD, HC + MD, HC + HD) for 4 weeks. We examined organ weight, conducted biochemical examinations of blood and urine, and analyzed the intestinal microbiota in feces through a 16s rDNA sequence analysis. In this study, TCI227 improved body weight, decreased creatinine and serum uric acid, and increased urine uric acid compared to the HC group. Furthermore, TCI227 increased short-chain fatty acids (SCFAs). In the fecal microbiota (family), TCI227 increased the level of *Lactobacillaceae* and then decreased the levels of *Deferribacteres* and *Prevotellaceae* compared to the HC group. Finally, in the fecal microbiota (genus), TCI227 decreased the level of *Prevotella* and then increased the levels of *Lactobacillus* and *Ruminococcus* compared to the HC group. This study suggested that TCI227 can improve HC and can change the composition of intestinal microbiota in PO-induced male HC SD rats.

## 1. Introduction

Uric acid (UA) is the final product of nucleic acid metabolism in the body [1]. Purine in the body is metabolized by the liver to form uric acid, and finally, the kidneys excrete uric acid from the body [2]. If too much UA is produced in the body or if it is poorly excreted by the kidneys, it forms crystals and that are deposited in the body, leading to hyperuricemia (HC), which is a chronic disease [3]. Risk factors include obesity, as well as the consumption of too much fructose, meat, or seafood [4]. HC patients are often accompanied by chronic diseases, such as gout, diabetes, heart disease, and kidney disease [5]. In addition to adjusting diet and weight control, the most direct way of treatment is drug intervention [6], but it has side effects, such as allergies, nephrotoxicity, liver toxicity, nausea, and vomiting, and most patients with HC are unable to achieve long-term control due to high-purine diets and poor treatment efficacy [7]. Therefore, in recent years, naturally derived dietary supplements are often used as preventive and adjuvant treatments for HC.

UA is secreted into the intestines and is rapidly metabolized by intestinal bacteria [8]. A study showed that the intestinal microbiota compositions of gout patients were significantly different compared to normouricemic subjects [8], suggesting that interaction among the microbiota, intestinal UA metabolism, and excretion can potentially modulate serum uric acid levels. A study also showed that changing the structure and amount of intestinal microbiota affected the metabolic rate of UA, thus reducing the UA content in the blood [9]. Furthermore, the transport protein of UA was found to be secreted by various indigenous microbes in the human gut [10]. Probiotic supplements have anti-uric acid, anti-inflammatory, immune regulation, and gastrointestinal barrier functions [11]. Therefore, the development of probiotic treatment strategies to reduce UA concentrations would be useful [11,12]. 

Probiotics are live microbial dietary supplements that exert beneficial effects on host health due to their potential effectiveness for the prevention and treatment of immune diseases [13]. Lactobacillus is the most common probiotic in the human body and is a kind of lactic acid bacteria. *L. plantarum* has a variety of potential beneficial effects on human health [14], such as improvement of the microbiota, immune regulation, suppression of fat accumulation, and reduction of serum triglycerides and cholesterol. Furthermore, *L. plantarum* treatment significantly reverses UA levels induced by a high-fat diet (HFD) and reduces the size of adipocytes [12,14]. However, it is still unclear whether *L. plantarum* can improve HC and intestinal microbiota.

Therefore, this study uses potassium oxonate (PO), a selectively competitive uricase inhibitor, to induce HC in rats [15] and then evaluates the effectiveness of *L. plantarum* TCI227 to improve HC and intestinal microbiota.

## 2. Materials and Methods

### 2.1. Animals and Ethics Statement

A total of forty-eight adult male SD rats (6 weeks old) were ordered for this study. All animals were kept in steel rodent cages, and the animal room temperature was controlled at 22 ± 2 °C. The humidity was controlled at 60–80%, with a 12 h light:dark cycle (light period: 07:00–19:00; dark period: 19:00–07:00). The animal use protocol listed below was reviewed and approved by the Chung Shan Medical University Institutional Animal Care and Use Committee (IACUC approval no: 2413). All animals were fed regular formula feed and distilled water until 8 weeks old; then, the test sample started the intervention.

### 2.2. Design of Animal Experiments

Forty-eight Wistar SD (Sprague–Dawley) rats were randomly assigned to 6 groups of 8 animals each. The control group was injected intraperitoneally with vehicle, while the others were injected intraperitoneally with a potassium oxonate suspension (Sigma-Aldrich, Schelldorf, Germany) of 250 mg/kg for hyperuricemia induction every day for 4 consecutive weeks. Test samples were administered during the hyperuricemia induction phenotype. The six groups were divided into: (a) the normal control group (control, C); (b) the hyperuricemia control group (hyperuricemia control, HC); (c) the low dose (10^8^ CFU/kg rat/day) of TCI227 oral gavage treatment hyperuricemia-induced group (low dose, LD) (HC + LD); (d) the medium dose (10^9^ CFU/kg rat/day) of TCI227 oral gavage treatment hyperuricemia-induced group (medium dose, MD) (HC + MD); (e) the high dose (10^10^ CFU/kg rat/day) of TCI227 oral gavage treatment hyperuricemia-induced group (high dose, HD) (HC + HD); and (f) the allopurinol (10 mg/kg rat/day) treatment hyperuricemia-induced group (HC + AP). From day 0 to day 28, the rats in groups (b)–(f) were treated once daily with PO, and those in groups (c)–(f) were treated with oral gavage of different doses of TCI227 and allopurinol. All animals were euthanized on day 28. On day 21, stool samples were collected from animals and kept in RNA stabilizer reagent (Cat. No. MLBRS0500, Taichung City, Taiwan) until day 28. The rats were fasted for 8–12 h before blood samples were collected to measure additional serum biochemical parameters from the tail veins prior to euthanization. Meanwhile, organ tissues, including heart, lung, liver, spleen, kidney, and adipose tissues (perirenal fat, epididymal fat, subcutaneous fat, mesenteric fat, and brown adipose tissue), as well as muscle tissues (gastrocnemius and soleus), were removed using PBS wash, and the weight was recorded prior to freezing with liquid nitrogen and storage at −80 °C.

### 2.3. Chemicals

Potassium oxonate (PO), allopurinol, and oxonic acid (potassium salt) were purchased from Sigma-Aldrich Chemie GmbH (Schnelldorf, Germany). *L. plantarum* powder (TCI227) was prepared by TCI Co. Ltd. A uric acid assay kit was purchased from Abcam (Cambridge, UK). Sodium chloride (NaCl) was purchased from Wako Pure Chemical Industries, Ltd. (Osaka, Japan). Formalin (HCHO) was purchased from Choneye Co., Ltd. (New Taipei City, Taiwan).

### 2.4. Serum Biochemical Parameter Analysis

Blood was collected in a serum separator tube (BD Vacutainer, Plymouth, UK) and centrifuged at 3000× *g* for 15 min, and then serum supernatant was transferred to the tube and stored at −80 °C for analysis. The levels of serum aspartate transaminase (AST), alanine transaminase (ALT), creatinine, blood urea nitrogen (BUN), triglycerides, total cholesterol, sodium ions (Na^+^), potassium ion concentration (K^+^), and chloride ions (Cl^−^) were analyzed with a fully automatic biochemical immunoassay analyzer (C501, Roche, Basel, Switzerland).

### 2.5. Uric Acid Content of Serum and Urine Analysis

Before the chemical intervention, blood samples were collected as control at week 0. Four weeks after intervention, blood samples were also collected for analysis. The blood was centrifuged at 3000× *g* for 15 min, and serum was taken out for analysis. Before the chemical intervention (week 0), as well as at the second and fourth weeks of the intervention, the rats were placed in a metabolic cage for 24 h, and their urine was collected in a centrifuge tube. Subsequently, the urine was centrifuged at 3000× *g* for 10 min at 4 °C, and the supernatant was collected. The levels of uric acid in the serum and urine were measured with a commercially available analysis kit (Abcam, Cambridge, UK).

### 2.6. Analysis of Short-Chain Fatty Acid Composition in Feces

A weight of 0.5 g of rat feces was added to 5 mL deionized water, homogenized for 2 min, and centrifuged at 7000 rpm for 5 min at 25 °C, and the supernatant was filtered. An amount of 1 mL supernatant was added to internal standard isocaproic acid, 100 μL 50% (*v*/*v*) sulfuric acid solution, and 1 mL of ether and homogenized for 2 min and centrifuged at 4000 rpm for 5 min at 25 °C. The supernatant was extracted. Then, it was further analyzed by gas chromatography (6890, Agilent Technologies, Santa Clara, CA, USA) for the contents of acetic acid, propionic acid, butyric acid, and valeric acid in the stool.

### 2.7. Stool DNA Purification and Next-Generation Sequencing for Intestinal Microbiota Analysis

The fecal nucleic acid extraction was purified using a QIAamp Fast DNA Stool Mini Kit to extract nucleic acid from bacteria in feces. Fecal samples were removed from the preservation solution at 13,200 rpm for 10 min. Feces were shaken and dissolved by adding InhibitEX buffer, and subsequent reagents were added in sequence. The supernatant was then collected and washed by transfer into a QIAmp spin column. Finally, preheated elution buffer was used for the elution of the DNA. A NanoDrop 2000 was used for concentration determination. Then, a next-generation sequence was used for further microbiota identification. The Illumina 16S Metagenomic Sequencing Library Preparation Manual Protocol was performed for intestinal microbiota analysis. The V3 and V4 gene fragments of bacterial 16S rRNA were amplified to develop amplicon libraries, and we further sequenced paired ends with Illumina MiSeq. A V3 MiSeq reagent kit (600 cycles) was used, with a sequencing quality of Q30 ≥ 80% and a single sample ≥100,000 reads.

### 2.8. Statistical Analyses

The experimental data were analyzed using Statistical Product and Service Solutions (SPSS), version 22.0. Variance analysis was performed using Student’s *t*-test with unpaired individuals, and the experimental data were presented as means ±SD, with a *p*-value < 0.05 considered statistically significant.

## 3. Results

### 3.1. TCI227 Improved Body Weight and Diet in PO-Induced HC Rats

**Figure A1:**
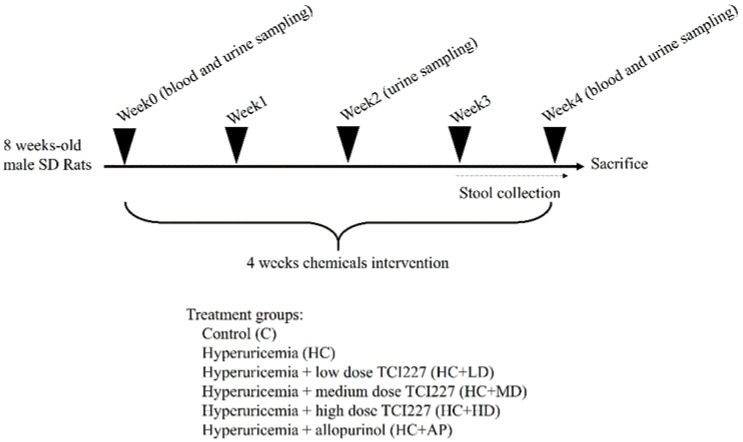
Schematic of probiotic-prevented hyperuricemia progression model.

To evaluate whether TCI227 improved HC and intestinal microflora, we used potassium oxonate (PO) (250 mg/kg, intraperitoneal injection) to induce HC in SD rats and then treated them with TCI227 in a dose-dependent manner (LD, MD, and HD) by oral administration for 4 weeks. Allopurinol can be used as an antihyperuricemia drug [16]. The detailed process is showed in Figure A1. We examined body weight and diet in PO-induced HC rats. Table 1 shows that there were no significant differences in initial body weight (week 0). However, the final weight (week 4) of the HC group was significantly decreased compared to that of the control group. Furthermore, the weight change in the HC group was significantly decreased compared with the control group, while the HC + MD and HC + HD groups were significantly increased compared with the HC group. The food intake of the HC group was significantly decreased compared with the control group, and the HC + LD, HC + MD, and HC + HD groups were significantly increased compared with the HC group. Water intake in the HC group was significantly decreased compared to the control group, and the HC + HD and HC + AP groups were significantly increased compared to the HC group. We also examined organ weight, adipose tissue weight, and muscle tissue weight in PO-induced HC rats.

Table 2 shows that the weights of the liver and spleen for the HC group were significantly increased compared to the control group in week 4, and the weight of the perirenal adipose tissue for the HC group was significantly decreased compared to the control group in week 4. There were no obvious changes in other organs and tissues. These results indicate that treatment with TCI227 could improve body weight and diet in PO-induced HC rats.

### 3.2. TCI227 Improved Hyperuricemia and Did Not Affect Liver and Kidney Function

Next, we examined blood and urine in PO-induced HC rats in week 4. Table 3 shows that there were no statistical differences between the groups in glucose, total cholesterol, AST, ALT, BUN, and potassium. Triglyceride and sodium ions in the HC group decreased significantly compared to the control group, and triglyceride in the HC + AP group decreased significantly compared to the HC group. Creatinine in the HC + MD group decreased significantly compared to the HC group, and chloride ions in the HC group increased significantly compared to the control group.

In addition, Table 4 shows that serum uric acid level in the HC group increased in week 4, while those of the HC + LD, HC + MD, and HC + AP groups decreased significantly compared to the HC group. The urine uric acid level in the HC group was decreased compared to the HC group in weeks 2 and 4, and those of the HC + LD and HC + MD groups were significantly increased compared to the HC group in week 4 and weeks 2 and 4, respectively. These results suggest that the administration of low (10^8^ CFU/kg/rat) and medium (10^9^ CFU/kg/rat) doses of TCI227 could decrease serum uric acid and increase urine uric acid, as well as that TCI227 did not influence liver and kidney function.

### 3.3. TCI227 Increased SCFAs and Diversity of Intestinal Microbiota

Studies had shown that intestinal bacteria can use dietary fiber to ferment into short-chain fatty acids (SCFAs), such as acetic acid, propionic acid, butyric acid, and valeric acid [17]. SCFAs can provide energy for intestine cells, maintain intestinal mucosa integrity, inhibit harmful bacteria, and promote the growth of beneficial bacteria [18]. Next, we examined fecal SCFAs in PO-induced HC rats. Table 5 shows that acetic acid, butyric acid, and valeric acid levels in the HC group were significantly decreased compared to the control group. SCFAs in the HC + LD, HC + MD, and HC + HD groups were increased more than those in the HC group. Then, we examined the diversity of the intestinal microbiota, and Shannon’s diversity index was used to estimate the level of community diversity [19].

**Figure 1:**
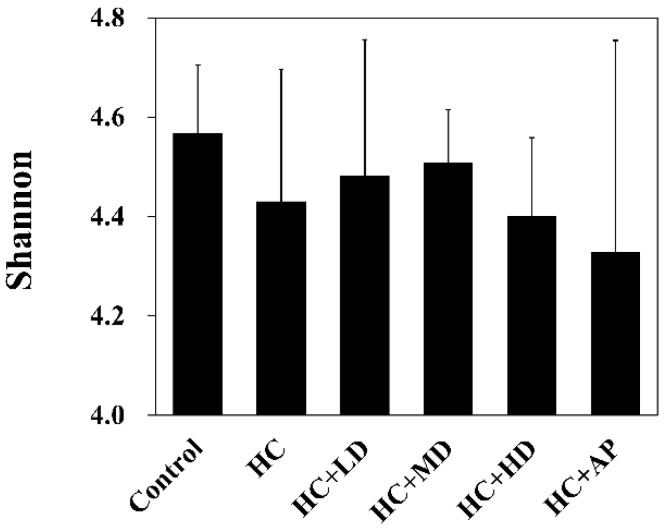
Alpha-diversity indices of richness of fecal microbiota compositions in rats with PO-induced hyperuricemia treated with TCI227. The Shannon index shows species richness and evenness. The reported values are means ± SD (n = 3). Significant difference among all the groups was not detected.

**Figure 2:**
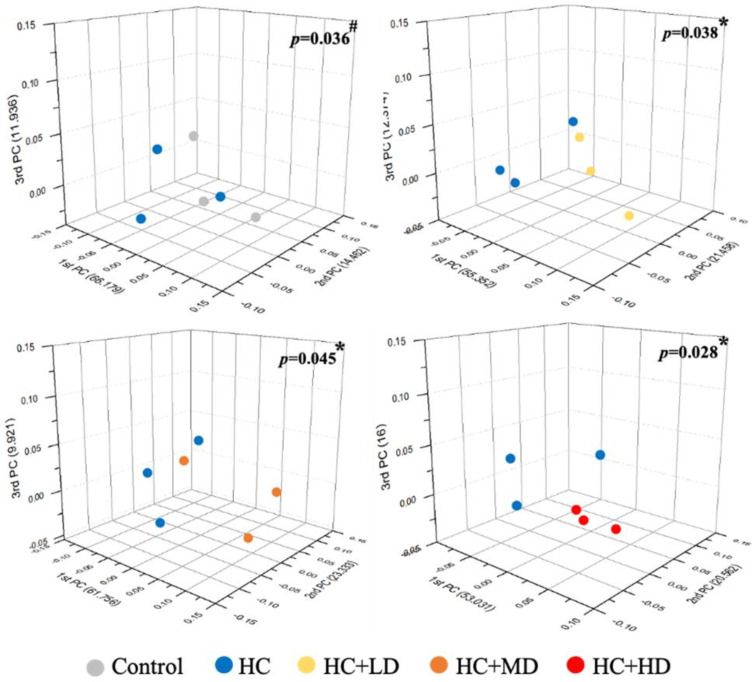
Effect of TCI227 on principal coordinates analysis (PCoA) index levels of fecal microbiota in rats with PO−induced hyperuricemia based on unweighted Jensen−Shannon distance measures of all samples based on OTU−level relative abundance profiles. Results are means for n = 3; ^#^ mean values are significantly different from control group (*p* < 0.05); * mean values are significantly different from HC group (*p* < 0.05) (Jensen−Shannon, genus, exclude unclassified OTU (reads)).

Figure 1 shows that the Shannon’s diversity index value for the HC group decreased slightly compared to the control group, and those of the HC + LD and HC + MD groups increased slightly compared to the HC group. Through a principal coordinates analysis (Figure 2), we found a significant difference between the HC group and the control group in microbiota composition and also found significant differences between the HC + LD, HC + MD, and HC + HD groups and the HC group. These results show that HC reduced SCFAs and the diversity of intestinal microbiota and that TCI227 could recover this phenomenon and regulate the composition of the microbiota.

### 3.4. TCI227 Changed Composition of Intestinal Microbiota

**Figure 3:**
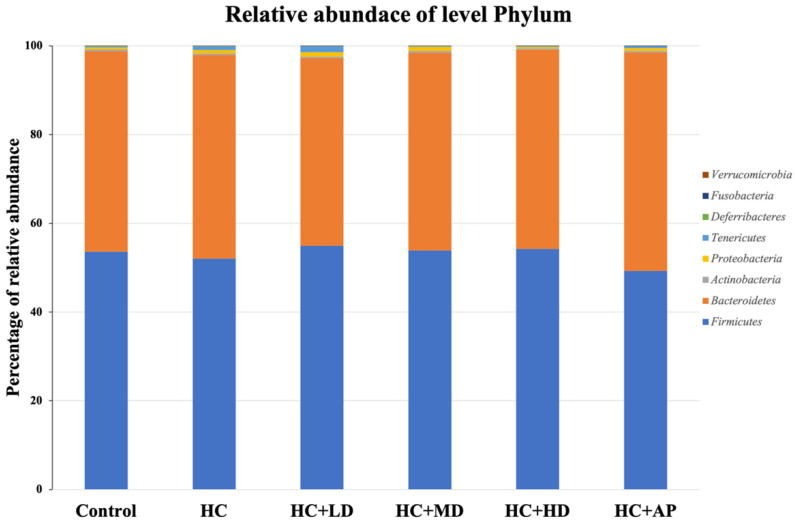
Effect of TCI227 on fecal microbiota phylum-level compositions in PO-induced hyperuricemia rats. The reported values are means (n = 3).

**Figure 4:**
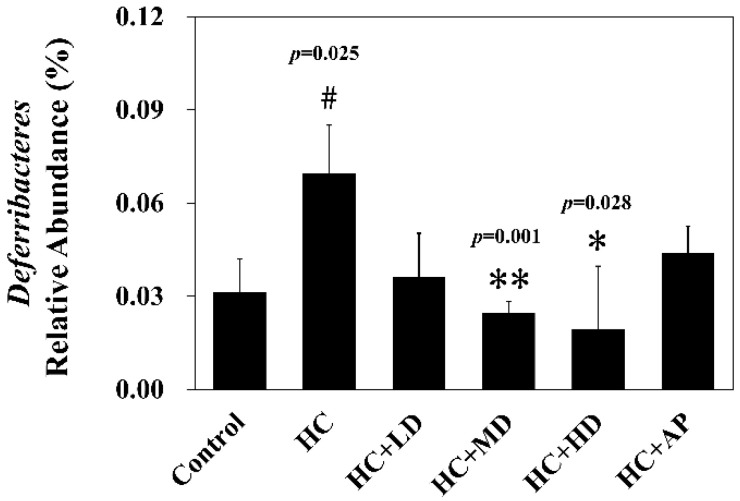
Effect of TCI227 on relative abundance of *Deferribacteres* in feces. The reported values are means ± SD (n = 3); # significantly differs from the HC group (*p* < 0.05); * significantly differs from the HC group (*p* < 0.05); ** significantly differs from the HC group (*p* < 0.01).

Next, we wanted to explore whether TCI227 could change the composition of intestinal microbiota using 16S microbiota ribosomal DNA sequencing. The eight most abundant fecal microbiota phylum of the CTL, HC, HC + LD, HC + MD, HC + HD, and HC + AP groups are shown (Figure 3). The most abundant microbiota (phylum) were *Firmicutes* and *Bacteroridetes.* When the ratio of *Firmicutes/Bacteroidetes* or the number of *Deferribacteres* are increased, it can promote obesity or gout in mice [20,21]. Figure 4 shows that the relative abundance of *Deferribacteres* in the HC group was significantly increased compared to the control group, while the HC+MD and HC+HD groups showed significantly decreased relative abundances of *Deferribacteres* compared to the HC group.

**Figure 5:**
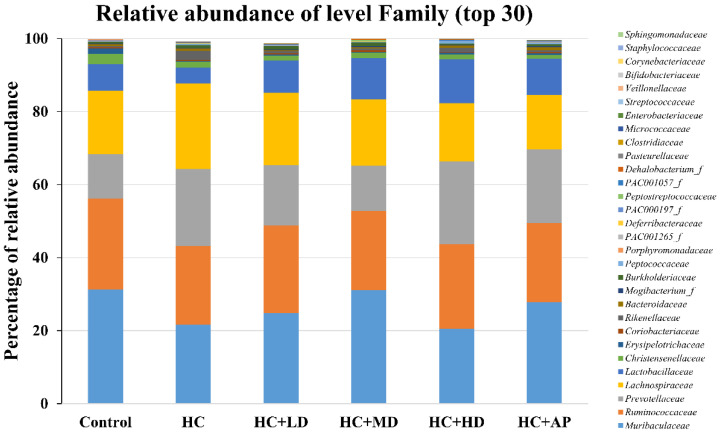
Effect of TCI227 on fecal microbiota family-level compositions in PO-induced hyperuricemia rats. The reported values are means (n = 3).

**Figure 6:**
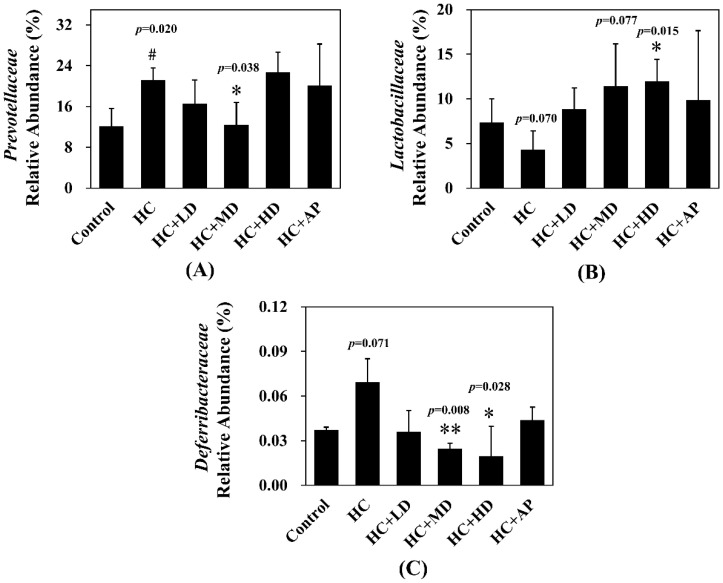
Effect of TCI227 on relative abundances of *Prevotellaceae* (**A**), *Lactobacillaceae* (**B**), and *Deferribacteraceae* (**C**) in feces. The reported values are means ± SD (n = 3); # significantly differs from the HC group (*p* < 0.05); * significantly differs from the HC group (*p* < 0.05); ** significantly differs from the HC group (*p* < 0.01).

In addition, the top 30 most abundant fecal microbiota families of the CTL, HC, HC + LD, HC + MD, HC + HD, and HC + AP groups are also shown (Figure 5). The most abundant microbiota (families) were *Muribaculaceaec* and *Ruminococcaceae.* Figure 6 shows that the relative abundance of *Prevotellaceae* in the HC group was significantly increased compared to the control group, and that of the HC + MD group was significantly decreased compared to the HC group. In addition, the relative abundance of *Lactobacillaceae* in the HC group decreased compared to the control group, and that of the HC + HD group was significantly increased. The relative abundance of *Deferribacteraceae* in the HC group was increased compared to the HC group, and those of the HC + MD and HC + HD groups were significantly decreased.

**Figure 7:**
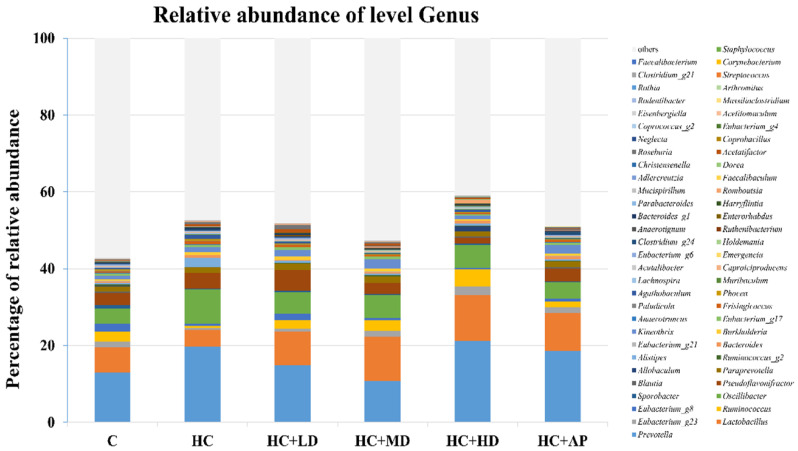
Effect of TCI227 on fecal microbiota genus-level compositions in PO-induced hyperuricemia rats. The reported values are means (n = 3).

**Figure 8:**
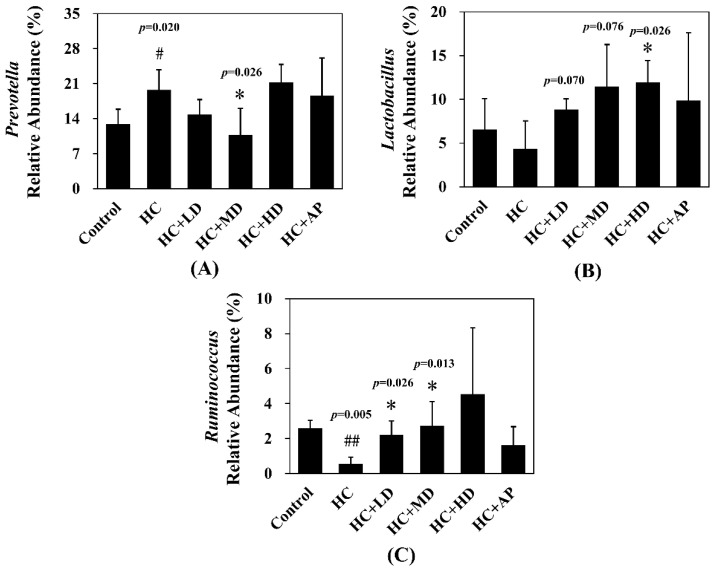
Effect of TCI227 on relative abundances of *Prevotella* (**A**), *Lactobacillus* (**B**), and *Ruminococcus* (**C**) in feces. The reported values are means ± SD (n = 3); # significantly differs from the HC group (*p* < 0.05); ## significantly differs from the HC group (*p* < 0.01); * significantly differs from the HC group (*p* < 0.05).

The major HC relative abundant fecal microbiota genera were *Prevotella*, *Oscillibacter*, and *Ruminococcus* (Figure 7). Figure 8 shows that the relative abundance of *Prevotella* in the HC group increased significantly compared to the control group, and that of the HC + MD group significantly decreased in abundance. The relative abundance of *Lactobacillus* in the HC group decreased in comparison to the control group, and that of the HC + HD group significantly increased in abundance. Moreover, the relative abundance of *Ruminococcus* in the HC group was significantly decreased compared to the control group, and those of the HC + LD and HC + MD groups were significantly increased in abundance. The above results indicate that TCI227 could change the composition of the intestinal microbiota.

## 4. Discussion

Most mammals contain uricase, which can metabolize uric acid as allantoin. However, several primates, including humans, have lost the function of uricase enzyme activity. It has been reported that uricase genes display two stop codons and act as a pseudogenes in human [22,23]. Without the metabolic ability of uric acid, elevated uric acid levels can cause the accumulation of urate crystals and lead to gout and gouty arthritis. In addition, hyperuricemia is associated with other metabolic diseases, including hyperlipidemia, hypertension, cardiovascular diseases, and diabetes [24]. In the past, antihyperuricemia drugs have been identified, including xanthine oxidase (XOD) inhibitors (allopurinol and febuxostat) and uricosuric agents (benzbromarone and probenecid), in clinical practice. Unfortunately, side effects limit the use of these drugs for hyperuricemia treatment [25,26]. Therefore, in this study, uricase inhibition modeled on potassium-oxonate-treated rats [27] was performed with a TCI227 probiotics intervention to investigate a new, improved strategy of hyperuricemia treatment.

A previous study showed that the body weight of a PO-induced hyperuricemia mouse model was significantly lower than that of control rats [28], which is similar to Table 1 of this study. More precisely, not only body weight, but also the results of food intake and water consumption records, were still within the physiological ranges of normal rats [29,30]. In the organ weight results of Table 2, the livers and spleens of PO-induced rats were significantly heavier than those of the control group (*p* < 0.05), and the weight of the perirenal fat tissue of PO-induced rats was significantly lighter than that of the control group (*p* < 0.05). We speculated two reasons for the increase in organ weight. First, the results of the histological section of the study revealed some necrosis in the peritoneal fat and an inflammation phenomenon after the intraperitoneal injection of PO (data not shown), which were probably associated with the formation of urate and could lead to foreign bodies, necrosis, and chronic granulomatous lesions. Second, the literature suggests that, when an organ develops a disease, the abundance of macrophages also increases [31]. Hence, after PO induced a hyperuricemia phenotype in the rats, an increase in the macrophages of organs could result in an increase in organ weight.

Regarding the toxicity test, a serum biochemical parameter analysis was performed, as shown in Table 3. The concentrations of serum triglycerides, creatinine, sodium ions, and chloride ions in the hyperuricemia groups were significantly different compared to the control group (*p* < 0.05). All of these chemical parameters were within the physiological ranges of normal rats in other research [32,33]. In brief, the TCI227 intervention did not cause liver injury, renal dysfunction, or electrolyte dysregulation. This evidence suggests that the TCI227 could be used as a safe oral health food to ameliorate hyperuricemia. 

According to Table 4, the low and medium doses of the TCI227 intervention could significantly reduce the serum uric acid concentrations of hyperuricemia rats (*p* < 0.05). Previous literature reports have also suggested that oral gavage probiotics of *Lactobacillus* (1.5 × 10^9^ CFU/mL/day) from kimchi or ursolic acid (5 and 10 mg/kg) can improve blood uric acid concentrations in PO-induced hyperuricemia rats [34,35]. Another study showed that, in PO-induced hyperuricemia mice, supplementation with Prunus mume fruit extract (140 mg/kg) for 1 week could increase urine uric acid excretion in mice [36]. This evidence suggests that supplementation with natural dietary supplements has the effect of ameliorating hyperuricemia. 

Recently, many chronic diseases (e.g., obesity, diabetes, and nonalcoholic fatty liver disease) have been associated with unbalanced dysregulation of intestinal flora-mediated inflammation [37]. A previous study indicated that hyperuricemia rats with high levels of long-term urate concentration in the blood had increased production of reactive oxygen species, leading to activated NF-κB or other responses from inflammation pathways. Subsequently, it promoted the expressions of inflammation factors IL-1β and TNF-α, causing immune dysfunction and leaky gut syndrome, followed by exaggerated immune response and intestinal barrier dysfunction, which triggered hyperuricemia progression [34,38].

A decrease in gut microbial diversity is always accompanied by an abnormal decrease in bacterial flora decrement and is associated with most human diseases [39]. Some studies have suggested that supplement intake has the effect of elevated microbial diversity. Figure 1 shows that PO-induced hyperuricemia rats had reduced abundances and α-diversity of the gut microbiota using Shannon’s diversity index analysis (*p* > 0.05). Medium and low doses of TCI227 intervention could slightly increase the Shannon’s diversity index values and improve the decrease in microbiota diversity decrement (*p* > 0.05). Although this result was not significant, the trend was very similar to a previous PO-induced hyperuricemia Kunming mice microbiota analysis. In that study, after a *Lactobacillus fermentum* JL-3 intervention, inflammatory markers and indicators of oxidative stress (IL-1β, MDA, CRE, and blood urea nitrogen) related to hyperuricemia were improved and microbial diversity was increased. Notably, another study showed that an allopurinol intervention did not significantly improve the diversity of gut flora (*p* > 0.05) in induced hyperuricemia SD rats on a high-fat diet containing 10% yeast extract, which is very similar to our results in Figure 1 [40]. 

In the analysis of the composition of microbiota phylum, Figure 4 suggests that the abundances of *Deferribacteres* were increased in PO-induced hyperuricemia rat fecal matter. According to a previous publication, an abundance of *Deferribacteres* was positively correlated with the concentration of IL-6 in plasma [41]. The pro-inflammatory factor IL-6 plays a crucial role in chronic inflammation diseases. Furthermore, past research showed that PO-induced hyperuricemia rats (250 mg/kg, rats) could display increases in IL-6 and other inflammation indicators [42]. In this study, the PO-induced hyperuricemia groups had higher abundances of *Deferribacteres* (*p* < 0.05). The medium and high doses of the TCI227 intervention significantly reversed the abundance of *Deferribacteres* (*p* < 0.05). *Deferribacteres* is correlated with immune response, which is positively associated with the levels of IL-6, IL-2, and TNF-α in serum. In a mouse model of sepsis treated with *Lactobacillus rhamnosus GG* (2 × 10^9^ CFU/mL) intestinal permeability and microbiota dysbiosis were improved. In that study, the abundance of *Deferribacteraceae* was reduced after *Lactobacillus rhamnosus GG* treatment [43]. In the microbiota family composition analysis, our results were similar to another group’s findings [44]. An intestinal dysbiosis phenomenon was observed in hyperuricemia-induced rats. The abundance of *Prevotellaceae* was increased in Figure 6, which is significantly similar to the TCI227 intervention in this study (*p* < 0.05). Furthermore, the abundance of *Lactobacillaceae* was reduced, which corresponds to an ICR mouse oral gavage of PO at 250 mg/kg from another group [45]. In our analysis of the composition of microbiota genera, the abundance of *Prevotella*, which is associated with chronic inflammation, significantly increased in hyperuricemia rats (*p* < 0.05). The intervention of TCI227 could significantly reverse the abundance of *Prevotella* (*p* < 0.05), as shown in Figure 7. According to a previous study [44], the abundances of *Lactobacillaceae* and *Rumminococcus* were decreased in hyperuricemia mice. Another study also suggested that the abundance of *Rumminococcus* decreased in mice inactivated with uricase [46]. Our results suggest that the abundances of *Lactobacillaceae* and *Rumminococcus* in hyperuricemia rats were lower than those of the control (*p* < 0.05), and this phenomenon was reversed by the TCI227 intervention. *Lactobacillaceae* and *Rumminococcus* can produce short-chain fatty acids (SCFAs), which can ameliorate kidney function. Hence, the increase in the abundances of *Lactobacillaceae* and *Rumminococcus* was facilitated by uric acid metabolism, and the intake of TCI227 could improve hyperuricemia.

A previous study suggested that intestinal microbiota could participate in purine and uric acid metabolism. On the other hand, SCFAs not only provided an energy resource for enterocytes, but also facilitated mucus secretion [47]. These bacterial fermentation products may play an important role in the mucoprotection of gut health by supporting the integrity of the mucosal barrier and preventing systemic inflammatory response. In hyperuricemia rats, less bacterial abundance of SCFA production was observed, including *Alistipes, Lactobacillus,* and *Ruminococcus*, resulting in low SCFA concentrations mediating intestinal barrier dysfunction and increasing intestinal permeability. In Table 5, the acetic acid, butyric acid, and valeric acid levels in the HC group were significantly lower than in the control group. After the TCI227 intervention, these SCFA contents were significantly increased (*p* < 0.05). No significant increase in SCFAs by the AP cotreatment group was derived due to the differently regulated mechanisms of AP and TCI227 in the amelioration of hyperuricemia.

## 5. Conclusions

Supplementation with TCI227 (low and medium doses of 10^8^ and 10^9^ CFU/kg rat, respectively) could ameliorate the concentration of uric acid in serum and the excretion of uric acid in urine in PO-induced hyperuricemia rats. In addition, administration of the TCI227 not only increased the abundance of intestinal microbiota, but also increased the concentrations of acetic acid, butyric acid, and valeric acid in PO-induced hyperuricemia rats. All these studies demonstrated that the administration of TCI227 improved hyperuricemia rats induced with potassium oxonate (PO).

## Figures and Tables

**Table 1:** Effects of TCI227 on the initial body weight, final body weight, weight change, food intake, and water intake of potassium-oxonate (PO)-induced hyperuricemia SD rats.

Groups	Control	HC	HC + LD	HC + MD	HC + HD	HC + AP
Initial body weight (g)	280.20 ± 13.00	292.94 ± 12.94	294.65 ± 17.29	294.30 ± 9.51	286.70 ± 19.66	287.20 ± 7.69
Final body weight (g)	399.83 ± 36.07	365.44 ± 21.92 ^#^	374.65 ± 13.55	388.24 ± 18.49	376.64 ± 25.09	374.04 ± 19.52
Weight change (g)	119.63 ± 24.63	72.50 ± 10.70 ^##^	80.00 ± 23.32	93.94 ± 19.49 *	89.94 ± 10.62 **	86.84 ± 17.80
Food intake (g/rat/day)	26.94 ± 0.86	22.68 ± 1.20 ^##^	25.12 ± 1.00 **	24.97 ± 1.11 **	24.45 ± 1.35 *	23.35 ± 1.03
Water intake (mL/rat/day)	45.66 ± 2.70	42.41 ± 2.92 ^#^	44.97 ± 4.20	42.44 ± 3.34	50.69 ± 2.68 **	47.43 ± 4.88 *

The reported values are means ± SD (n = 8); # significantly differs from the HC group (*p* < 0.05); ## significantly differs from the HC group (*p* < 0.01); * significantly differs from the HC group (*p* < 0.05); ** significantly differs from the HC group (*p* < 0.01). HC, hyperuricemia control; LD, low dose (10^8^ CFU/kg rat); MD, medium dose (10^9^ CFU/kg rat); HD, high dose (10^10^ CFU/kg rat); AP, allopurinol. Weight change (g) = final body weight (g)—initial body weight (g).

**Table 2:** Effects of TCI227 on the weights of organs, adipose tissues, and muscle tissues in PO-induced hyperuricemia SD rats.

Weight of Organs and Tissues (mg/g rat)	Control	HC	HC + LD	HC + MD	HC + HD	HC + AP
Liver	33.62 ± 1.74	38.56 ± 1.57 ^##^	38.29 ± 2.07	38.79 ± 2.47	37.08 ± 2.33	37.06 ± 2.19
Heart	3.53 ± 0.18	3.54 ± 0.26	3.58 ± 0.25	3.48 ± 0.30	3.49 ± 0.38	3.41 ± 0.23
Lung	3.72 ± 0.32	3.99 ± 0.23	3.82 ± 0.37	3.83 ± 0.19	4.04 ± 0.19	4.05 ± 0.40
Spleen	2.36 ± 0.92	5.66 ± 1.00 ^##^	6.52 ± 0.81	5.78 ± 1.05	5.27 ± 1.14	5.52 ± 1.14
Kidney	8.43 ± 1.00	8.51 ± 0.49	8.45 ± 0.64	9.00 ± 0.48	8.49 ± 0.89	8.11 ± 0.38
Epididymal adipose tissue	8.89 ± 1.67	7.52 ± 1.64	8.51 ± 1.95	6.97 ± 1.49	7.83 ± 1.62	6.77 ± 3.16
Perirenal adipose tissue	11.05 ± 3.04	7.52 ± 2.25 ^#^	8.87 ± 3.39	7.31 ± 1.58	8.02 ± 2.62	7.39 ± 1.46
Mesenteric adipose tissue	6.00 ± 2.21	7.44 ± 2.68	10.12 ± 2.83	10.31 ± 5.75	9.05 ± 3.26	7.69 ± 2.21
Subcutaneous adipose tissue	13.28 ± 2.94	11.07 ± 2.98	12.74 ± 3.32	11.50 ± 1.90	12.75 ± 1.94	9.44 ± 1.76
Brown adipose tissue	0.64 ± 0.20	0.52 ± 0.10	0.45 ± 0.22	0.61 ± 0.28	0.49 ± 0.16	0.58 ± 0.17
Gastrocnemius muscle	7.87 ± 2.83	9.21 ± 2.49	9.14 ± 2.67	8.71 ± 2.66	9.11 ± 2.85	9.16 ± 2.38
Soleus muscle	0.92 ± 0.14	0.84 ± 0.08	0.84 ± 0.10	0.83 ± 0.11	0.90 ± 0.09	0.91 ± 0.07

The reported values are means ± SD (n = 8); # significantly differs from the HC group (*p* < 0.05); ^##^ significantly differs from the HC group (*p* < 0.01).

**Table 3:** Effects of TCI227 on serum biochemical parameters in PO-induced hyperuricemia SD rats.

Groups	Control	HC	HC + LD	HC + MD	HC + HD	HC + AP
Glucose (mg/dL)	97.25 ± 9.95	97.75 ± 12.96	89.13 ± 5.74	97.38 ± 19.04	90.13 ± 8.59	91.13 ± 10.88
Total cholesterol (mg/dL)	44.88 ± 5.87	46.00 ± 9.59	44.75 ± 11.46	48.88 ± 7.62	48.38 ± 8.45	40.63 ± 8.23
Triglyceride (mg/dL)	83.75 ± 29.50	50.88 ± 12.18 ^#^	45.00 ± 13.66	43.75 ± 14.32	42.38 ± 10.68	37.50 ± 5.86 *
AST (U/L)	75.50 ± 5.63	77.25 ± 14.83	93.88 ± 36.76	69.63 ± 13.54	77.75 ± 14.44	73.63 ± 11.21
ALT (U/L)	38.88 ± 6.85	30.13 ± 9.39	36.50 ± 6.30	31.50 ± 8.80	37.50 ± 15.86	30.50 ± 8.77
BUN (mg/dL)	14.38 ± 1.30	14.13 ± 1.89	14.38 ± 2.88	13.25 ± 1.83	14.75 ± 2.92	16.50 ± 4.44
Creatinine (mg/dL)	0.35 ± 0.05	0.34 ± 0.04	0.32 ± 0.06	0.28 ± 0.03 **	0.31 ± 0.03	0.34 ± 0.05
Na^+^ (mmol/L)	145.25 ± 1.67	141.75 ± 1.75 ^##^	142.38 ± 1.06	141.25 ± 1.58	141.38 ± 2.20	144.00 ± 1.85
K^+^ (mmol/L)	6.36 ± 0.17	6.64 ± 0.67	6.84 ± 0.71	6.84 ± 0.45	6.74 ± 1.01	6.48 ± 0.50
Cl^−^ (mmol/L)	96.76 ± 1.38	99.05 ± 2.71 ^#^	99.45 ± 2.41	98.49 ± 1.39	98.70 ± 1.93	100.15 ± 0.98

The reported values are means ± SD (n = 8); ^#^ significantly differs from the HC group (*p* < 0.05); ## significantly differs from the HC group (*p* < 0.01); * significantly differs from the HC group (*p* < 0.05); ** significantly differs from the HC group (*p* < 0.01).

**Table 4:** Effects of TCI227 on serum uric acid and urine uric acid in PO-induced hyperuricemia SD rats.

Groups	Control	HC	HC + LD	HC + MD	HC + HD	HC + AP
Serum uric acid (mg/dL)						
Week 0	4.05 ± 1.70	4.41 ± 1.71	4.11 ± 2.46	4.04 ± 2.77	4.08 ± 1.68	4.27 ± 2.25
Week 4	4.83 ± 0.70	6.52 ± 0.91 ^##^	4.96 ± 1.77 *	5.16 ± 1.54 *	5.22 ± 1.80	1.32 ± 0.76 **
Urine uric acid (mg/dL)						
Week 0	7.41 ± 3.03	7.68 ± 3.28	6.55 ± 0.54	7.70 ± 2.81	7.86 ± 3.53	7.64 ± 2.53
Week 2	6.66 ± 1.78	3.14 ± 2.22 ^##^	4.48 ± 2.51	5.21 ± 2.86 **	5.59 ± 2.78	3.23 ± 1.61
Week 4	6.39 ± 1.42	3.91 ± 1.71 ^##^	6.08 ± 1.72 *	5.41 ± 0.72 *	5.15 ± 0.69	4.71 ± 1.67

The reported values are means ± SD (n = 8); ## significantly differs from the HC group (*p* < 0.01); * significantly differs from the HC group (*p* < 0.05); ** significantly differs from the HC group (*p* < 0.01).

**Table 5:** Effects of TCI227 on fecal short-chain fatty acids (SCFAs) in PO-induced hyperuricemia SD rats.

Groups	Control	HC	HC + LD	HC + MD	HC + HD	HC + AP
Acetic acid (μmol/g)	8.22 ± 1.73	5.21 ± 0.50 ^##^	7.17 ± 2.31	5.56 ± 1.71	5.34 ± 0.60	4.67 ± 1.23
Propionic acid (μmol/g)	2.62 ± 0.98	1.79 ± 0.43	2.30 ± 0.77	1.97 ± 0.70	1.97 ± 0.22	1.84 ± 0.52
Butyric acid (μmol/g)	8.60 ± 4.38	4.14 ± 1.29 ^#^	5.41 ± 1.80	5.68 ± 5.30	4.34 ± 1.18	4.69 ± 3.46
Valeric acid (μmol/g)	0.77 ± 0.21	0.50 ± 0.12 ^#^	0.50 ± 0.15	0.55 ± 0.20	0.51 ± 0.04	0.46 ± 0.13

The reported values are means ± SD (n = 6); # significantly differs from the HC group (*p* < 0.05); ^##^ significantly differs from the HC group (*p* < 0.01).

## Data Availability

The datasets analyzed or generated during this study are available from the corresponding author on reasonable request.
